# Extraction, Identification, and In Vitro Anti-Inflammatory Activity of Feruloylated Oligosaccharides from Baijiu Distillers’ Grains

**DOI:** 10.3390/foods13081283

**Published:** 2024-04-22

**Authors:** Zhongtian Yin, Mengyao Liu, Bowen Wang, Dongrui Zhao, Hehe Li, Jinyuan Sun

**Affiliations:** 1 Beijing Advanced Innovation Center for Food Nutrition and Human Health, College of Food Science and Nutritional Engineering, China Agricultural University, Beijing 100083, China; b20193060516@cau.edu.cn; 2 Key Laboratory of Geriatric Nutrition and Health, Ministry of Education, Beijing Technology and Business University, Beijing 100048, China; lmy040024@163.com (M.L.); wangbw@btbu.edu.cn (B.W.); zdr@btbu.edu.cn (D.Z.); lihehe@btbu.edu.cn (H.L.)

**Keywords:** feruloylated oligosaccharides, extraction, structure, antioxidant, anti-inflammatory

## Abstract

The structure and function of phenoyl oligosaccharides in baijiu distillers’ grains (BDGs) have not been identified and investigated yet. This study aimed to elucidate the major phenolic oligosaccharides present in BDGs, optimize their extraction process via a central composite design, and assess their anti-inflammatory properties utilizing the LPS-induced RAW264.7 inflammation model. The main results are as follows: feruloylated oligosaccharides (FOs) were identified as the main phenoyl oligosaccharides in BDGs with a structure of ferulic acid esterified on arabinooligosaccharide xylose. Then, the preparation process of FOs was optimized using the following conditions: pH 5, temperature 55 °C, time 12 h, xylanase addition amount 7 g/L, BDG concentration 120 g/L. Furthermore, the acquired FOs demonstrated notable scavenging activity against DPPH and ABTS free radicals, with Trolox equivalent values of 366.8 ± 10.38 and 0.35 ± 0.01 mM Trolox/mg sample, respectively. However, their efficacy was comparatively lower than that of ferulic acid. Finally, the obtained FOs could effectively inhibit the LPS-induced secretion of TNF-α, IL-6, and IL-1β and promote the secretion of IL-10 in RAW264.7 cells. Based on the above results, FOs from BDGs were determined to have certain antioxidant and anti-inflammatory activities.

## 1. Introduction

Phenoyl oligosaccharides (POs) constitute natural bioactive compounds resulting from the bonding of phenolic acids with oligosaccharides primarily found within the cell walls of diverse grass species [1]. According to relevant reports, at the cellular level, feruloylated oligosaccharides (FOs) exhibit a concentration-dependent inhibition of DNA damage in human lymphocytes induced by 100 μM H_2_O_2_; feruloylated arabinoxylan from wheat bran demonstrates inhibitory effects on M1-macrophage activation and promotes M2-macrophage polarization [2,3]. In animal experiments, the oral administration of FOs extracted from corn bran significantly influences the immunoregulation of murine colitis, restoring the immune balance between Th17 and Treg cells [4]. Additionally, POs exhibits diverse activities, including anti-glycosylation, antioxidant, and probiotic effects [5], and these functional activities cover almost both ferulic acid and oligosaccharides.

An investigation into natural phenolic acids revealed that, in plants, phenolic acids typically reside in the collateral branches of polysaccharides. As POs was released from the polysaccharide chain, its biological activity was gradually discovered [5]. FOs, as a typical POs characterized by ester bonds between the carboxyl group of ferulic acid and the hydroxyl group of oligosaccharides, have emerged as a focal point in recent POs research. Currently, FOs extraction methods include acid hydrolysis, enzymatic methods, and physical methods, among others [6,7,8]. Acid hydrolysis releases POs by hydrolyzing plant cell wall polysaccharides with chemical reagents, albeit with potential hazards to human health due to the use of strong acid reagents. Enzymatic hydrolysis, utilizing xylanase, cellulase, or breakdown enzymes, presents milder reaction conditions that preserve the structure of POs. Notably, POs has been discovered and utilized in the redevelopment of agricultural by-products such as corn bran, wheat bran, and brewers spent grain, contributing to the sustainable utilization of agricultural residues [9,10,11].

BDGs constitute the residual material after distilling fermented grains, primarily comprising sorghum bran and other brewing raw materials [12]. Only a small part of the distillers’ grains generated by each round of steaming baijiu may enter the next brewing round, while the accumulation of the remaining distillers’ grains causes environmental pollution [13]. As a major byproduct of baijiu production, over ten million tons of BDGs are generated annually, necessitating production enterprises’ environmentally sustainable management [14]. Rational treatment of BDG has gradually become an important link in the green development of the baijiu industry. According to reports, various bioactive substances have been found in BDGs, such as phenolic acid, active peptides, proteins, and polysaccharides [15,16,17]. The utilization of PO in BDGs represents the simultaneous application of phenolic acid and oligosaccharides, streamlining the treatment process and enhancing the value of BDGs. However, different from other raw materials, whether PO from BDGs possesses multiple biological activities needs to be further studied.

RAW264.7 macrophages, commonly employed as immune cells in inflammatory cell models, exhibit an overexpression of immune response markers such as NO, TNF-α, IL-1β, and IL-6 under Lipopolysaccharide (LPS) induction [18]. Compounds capable of inhibiting the expression of inflammatory factors in RAW264.7 cells may be developed as effective anti-inflammatory inhibitors. Consequently, this paper presents a study that aimed to optimize the process of extracting PO from BDGs, identify its structure, evaluate its in vitro antioxidant activity using DPPH and ABTS assays, and assess its anti-inflammatory activity through an LPS-induced RAW264.7 cell inflammation model. This study endeavors to unravel the antioxidant and anti-inflammatory activities of PO derived from BDGs, providing insights for the high-value utilization of this baijiu byproduct.

## 2. Materials and Methods

### 2.1. Materials and Reagents

BDGs, the residual bran obtained subsequent to the distillation of fermented sorghum, rice, wheat, glutinous rice, and maize materials, were generously supplied by Gujing Group Co., Ltd. (Bozhou City, Anhui Province, China). Sampling Methodology: sampling was conducted at three tiers (upper, middle, and lower) within the fermentation vat, ensuring a minimum of five sampling points per tier. At each sampling point, a sample weighing of 200 g was collected. Following thorough mixing, the distillers’ grains were piled into a conical shape. Subsequently, the “cone quartering method” was employed for sampling in preparation for subsequent analysis. The samples underwent dehydration at 50 °C and were subsequently sieved through a 40-mesh filter, yielding the BDG stive, which was then stored at −20 °C.

Neutral protease (50 U/mg), α-amylase (40 U/mg), xylanase (60,000 U/g), glucosidase (100,000 U/mL), ferulic acid standards, XAD-2 macroporous resin, DPPH radical, and ABTS radical were purchased from Macklin Biochemical Technology Co. (Shanghai, China). Trolox, ferulic acid, mannose, ribose, rhamnose, glucuronic acid, galacturonic acid, N-acetylglucosamine, glucose, N-acetylgalactosamine, galactose, xylose, arabinose, and fucose were purchased from Sigma-Aldrich Chemical Co. (Shanghai, China). Dulbecco’s Modified Eagle’s Medium (DMEM) and Fetal Bovine Serum (FBS) were sourced from Gibco Invitrogen Corporation, while LPS was procured from Beyotime Biotechnology. All other chemicals and reagents utilized throughout the study adhered to analytical-grade standards, with a minimum purity of 99%.

### 2.2. Identification of Phenolic Acids in BDGs

The types of phenolic acids in BDGs were preliminarily identified [19]. The process was as follows: BDG (2 g) was added to 20 mL 2 M NaOH in 60 °C water bath for 2 h. Subsequently, the reaction system was adjusted to pH 5 using 2 M HCl, followed by the liquid–liquid extraction of ferulic acid with the addition of 5 mL of ethyl acetate. The upper layer of the solution was aspirated, transferred to a glass culture dish, and desiccated in an oven at 50 °C. The dish was further rinsed with 1 mL of methanol and transferred over a 0.45 μm membrane to an injection bottle for subsequent analysis by high-performance liquid chromatography (HPLC) using an Agilent (Santa Clara, CA. USA) 1260 system in series with a Xbridge Peptide BEH C18 column (150 mm × 4.6 mm; 5 µm). The chromatographic conditions were configured as follows: Solvent A consisted of 0.1% formic acid in ultrapure water, while Solvent B comprised chromatographic-grade methanol. The gradient elution program involved 95% to 60% A over 0–10 min and 60% to 40% A over 10–15 min. The elution process was carried out at a flow rate of 1 mL/min and a column temperature maintained at 25 °C. UV absorption at a wavelength of 322 nm was monitored using a VWD detector for analysis.

### 2.3. Extraction of FOs from BDGs

The FO extraction method used was based on the one described in [20], with some modifications. A total of 100 g of BDG was homogenously mixed with deionized water at a material-to-liquid ratio of 1:10 (*w*/*v*). Subsequently, α-amylase and glucosidase were introduced, and the mixture underwent hydrolysis in a 55 °C water bath for 2 h, followed by inactivation at 95 °C for 10 min. The pH of the reaction system was then adjusted to 7 using NaOH. Neutral protease was added, and the reaction proceeded in a 50 °C water bath for 2 h, followed by inactivation at 95 °C. The supernatant was separated through centrifugation (8000 rpm, 10 min), and the residue was reconstituted with deionized water. Xylanase was introduced, and magnetic stirring ensued in a 60 °C water bath for 6 h. The resulting supernatant was obtained through centrifugation (8000 rpm, 10 min).

The obtained supernatant was subjected to loading onto XAD-2 macroporous resin. The resin column underwent initial rinsing with three column volumes of deionized water to eliminate polysaccharides devoid of ferulic acid. Subsequently, FOs were washed with a 50% ethanol aqueous solution, and ethanol was removed from the fraction via rotary evaporation. The resultant liquid was finally filtered through a Milli-Q ultrafiltration system (equipped with 3000 Dalton ultrafiltration membrane), and the FO powder was acquired through freeze-drying.

### 2.4. Response Surface Optimization of Extraction Process

The investigation into the single-factor impact on FOs in BDG production commenced with a systematic exploration of each factor to establish the optimal level interval for every variable. Subsequently, a comprehensive optimization of FO extraction conditions was undertaken using a five-variable and five-level central composite design. The reaction system was constructed within a 10 mL centrifuge tube. The entire process was executed, and FO production was quantified by the UV absorbance value at 322 nm of an equivalent volume of supernatant. The absorbance value served as the *Z* axis, with the factor levels on the *X* and *Y* axes forming the response surface graph. The vertex of the response surface graph represented the maximum yield achievable under the optimal variable conditions. 

The five factors subjected to optimization encompassed pH, substrate concentration, enzyme addition amount, time, and reaction temperature, with specific factor levels outlined in Supplementary Table S2, in which the five levels of each variable are represented by −2, −1, 0, 1, and 2, indicating the lowest, medium–low, medium, high, and highest levels, respectively.

### 2.5. Characterization of FOs

#### 2.5.1. Determination of the Degree of Substitution

The determination of the degree of substitution (DS) of ferulic acid followed established methodologies referenced in previous reports, such as the method in [21]. The quantitative analysis of ferulic acid was carried out by HPLC using the external standard method, and the HPLC method utilized in this subsection corresponds to the procedure described in Section 2.2. Additionally, standard ferulic acid was dissolved in chromatographic-grade methanol and diluted to gradient concentrations, followed by HPLC analysis in the same manner as for the samples. The linearity of the calibration regression line was R^2^ = 0.9948. The equation of the standard curve of ferulic acid was y = 30.994x + 7.3187. The calibration curve equation of ferulic acid was y = 30.994x + 7.3187. The linearity ranged from 1 to 1000 mg/L. The limit of detection (LOD) was 0.051 mg/L with a signal-to-noise ratio of 3:1, and the limit of quantification (LOQ) (10:1) was 0.165 mg/L. The calculation of the degree of substitution (DS) followed the formula outlined in Equation (1):

(1) $$DS=\frac{150\times W}{100\times 194.19-(194.19-1)\times W}$$

where 150 is the molecular weight of the xyloaglycone unit; 194.19 is the molecular weight of the ferulic acid; and W is the percentage of ferulate in the sample.

#### 2.5.2. Analysis of UV–Visible Transmission Spectra and Fourier-Transform Infrared Spectroscopy

FOs were solubilized in ultrapure water and subjected to spectral analysis within the wavelength range of 190–400 nm using a UV–visible spectrophotometer. Subsequently, Fourier-transform infrared spectroscopy (FT-IR) was employed to conduct a comprehensive scan of FOs within the wavenumber range of 600–4000 cm^−1^.

#### 2.5.3. Analysis of Monosaccharide Composition

The composition of monosaccharides was determined according to the method reported in [22], with some modifications. A total of 1 mg of FOs was dissolved in 3 mL of 2 M trifluoroacetic acid and introduced into an ampoule filled with nitrogen gas, which was subsequently sealed and subjected to acidification for 4 h at 120 °C. Following this, methanol was introduced into the ampoule, and nitrogen blowing was applied until the solvent evaporated. The residue was then reconstituted in 3 mL of water. In an EP tube, 250 µL of the sample solution was combined with 250 µL of 0.6 M NaOH and 500 µL of 0.4 M PMP (dissolved in methanol). The reaction transpired at 70 °C for 1 h, succeeded by cooling in cold water for 10 min and neutralization with 500 µL of 0.3 M HCl. Finally, 1 mL of chloroform was added for vortexing, followed by centrifugation for 10 min, and then, the supernatant was collected. This extraction process was iterated thrice. 

The monosaccharide composition analysis was conducted using a Shimadzu LC-20AD, employing an Xtimate C18 column (4.6 × 200 mm, 5 µm) for component separation. The column temperature was maintained at 30 °C, and detection occurred at 250 nm. An equal degree elution procedure was employed, utilizing a mobile phase consisting of a 0.05 M potassium dihydrogen phosphate aqueous solution (pH adjusted to 6.7 with NaOH) and acetonitrile (83:17). A total of 1 mg of various monosaccharides, including mannose, ribose, rhamnose, glucuronic acid, galacturonic acid, N-acetylglucosamine, glucose, N-acetylgalactosamine, galactose, xylose, arabinose, and fucose, was accurately weighed and then dissolved in 1 mL of ultrapure water and diluted to 50 μg/mL. Monosaccharides were derivatized in the same way as the samples to be tested. The resulting standard curves, depicted in Supplementary Table S1, exhibited exceptional linearity across the entire concentration range of standard monosaccharides, with a correlation coefficient (R^2^) exceeding 0.9910.

### 2.6. In Vitro Antioxidant Activity of FOs

The in vitro antioxidant capacity of FOs was assessed according to their ability to neutralize free radicals through two distinct methods: DPPH free radical scavenging and ABTS free radical scavenging. Ferulic acid served as the positive control group and was dissolved in 50% ethanol, with final concentrations of 12.5, 25, 50, 125, 250, and 500 mg/L. The method for determining the ability of FOs to clear DPPH free radicals was based on that in a previous report and partially modified [23]. FOs were dissolved in 50% chromatographic-grade ethanol at concentrations of 125, 250, 500, 1250, 2500, and 5000 mg/L. Subsequently, 100 µL of the tested FOs solution was added to a 96-well plate, followed by the addition of 100 µL of 0.1 M DPPH (dissolved in methanol). The 96-well plate was then incubated at room temperature in the dark for 30 min, and absorbance was measured at 517 nm using a microplate reader. The DPPH radical scavenging rate was calculated as per Equation (2):

(2) $$DPPH\;radical\;scavenging\;rate\;(\%)=\left[1-\frac{A_{1}-A_{2}}{A_{0}}\right]\times 100\%$$

where A_0_ is the absorbance value of the blank control (ultrapure water), A_1_ is the absorbance value of the sample with DPPH solution, and A_2_ is the absorbance value of the sample with ultrapure water. 

The method used for determining the effect of FOs on ABTS radical scavenging referred to the method reported in [24]. Briefly, the ABTS free radical solution was initially prepared and pH-adjusted to 7.4 using a 0.2 M phosphate-buffered solution, ensuring an absorbance of 0.7 ± 0.02 at 734 nm. Subsequently, 30 µL of the test sample was combined with 170 µL of the ABTS solution in a 96-well plate and allowed to incubate at room temperature in the dark for 6 min. Finally, the absorbance value was measured at 734 nm.

Ferulic acid was employed as the positive control, and the Trolox equivalent value (mg sample/mM Trolox) was determined by selecting the concentration associated with the maximum clearance rate within the concentration range proportional to the free radical elimination rate.

### 2.7. FOs Alleviate LPS-Induced Inflammation in RAW 264.7 Cells

#### 2.7.1. Incubation of RAW 264.7 Cells

The RAW 264.7 murine macrophage cell line was acquired from the China Type Culture Collection Center (Wuhan, China). These cells were cultured in high-glucose DMEM supplemented with 10% FBS and 1% antibiotics within a culture flask. The cell culture conditions were maintained in a controlled environment with 95% air and 5% CO_2_ at a temperature of 37 °C.

#### 2.7.2. LPS-Induced RAW 264.7 Cell Inflammation Model

Upon achieving a cell confluence exceeding 90% of the culture bottle’s surface area, the adherent cells were detached using a cell scraper and subsequently transferred to a centrifuge tube. The cell culture medium was then diluted to a concentration of 1 × 10^5^ cells/mL with DMEM medium and transferred to a 24-well plate at 900 µL per well. The plate was then placed in a culture incubator for a 24 h incubation period. Following incubation, the sample group was subjected to the addition of 100 µL of Lipopolysaccharide (LPS) at concentrations of 0, 25, 50, 100, 300, 600, and 900 mg/L to each well, and optimization experiments were conducted for FO dosing concentrations. In the blank group, 100 µL of DMEM culture medium was added to each well, while in the sample group, 100 µL of FOs at different concentrations (dissolved in DMEM as the solvent: 2.5, 5, 10, 25, 50, 125, and 250 mg/mL) was added to each well. The samples were further incubated in the culture incubator for an additional 24 h. For each concentration group, the procedure was replicated in triplicate wells. Finally, cellular viability was assessed using the CCK-8 assay kit.

#### 2.7.3. Determination of Inflammatory Factors in RAW 264.7 Cells

After the cells were transferred into a 24-well plate (800 µL/well), the blank control group received an additional 200 µL of DMEM medium. The model group was treated with 100 µL of Lipopolysaccharide (LPS) and 100 µL of DMEM medium per well. In the sample group, each well received 100 µL of LPS and 100 µL of FOs, with three replicates established for each sample. The secretion of nitric oxide (NO) was assessed using an NO kit, while the concentrations of IL-6, IL-1β, TNF-α, and IL-10 were determined through using the corresponding enzyme-linked immunosorbent assay (ELISA) kits. The protein concentration of the cells was quantified using the Bicinchoninic Acid kit.

### 2.8. Statistical Analysis

Statistical analysis was conducted using GraphPad Prism 9.5 (GraphPad Software Inc., San Diego, CA, USA). Data are reported as the mean ± SD and were assessed for normal distribution and similar variance between groups. Statistical significance (* *p* < 0.05, ** *p* < 0.01, *** *p* < 0.001, **** *p* < 0.0001) was determined using a one-way analysis of variance (ANOVA) with Tukey’s post hoc tests for multiple comparisons.

## 3. Results

### 3.1. Preparation of PO

**Figure 1:**
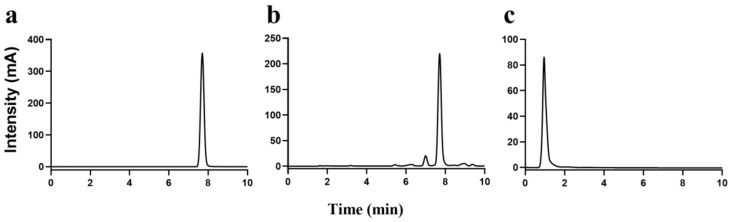
Identification of phenolic acids and FOs in distillers’ grains by HPLC. (**a**) Ferulic acid standards; (**b**) ferulic acid isolated upstream from FOs; (**c**) FOs.

The identification of phenolic acid types in BDGs is essential prior to the establishment of an effective extraction method, ensuring both efficiency and specificity in the extraction of PO. The HPLC results, depicted in Figure 1a,b, indicated a retention time of 7.7 min for the ferulic acid standard. Following the hydrolysis of ester bonds in FOs by NaOH, ferulic acid was liberated, aligning its retention time with the ferulic acid standard. Although minor chromatographic peaks were observed, their low content leads to the conclusion that the primary constituent in the sample is ferulic acid-bound oligosaccharides. Consequently, it can be inferred that the phenolic compounds in BDG are predominantly feruloyl polysaccharides. In accordance with a prior study [25], the presence of ferulic acid in plant cell walls is mainly attributed to FOs. Therefore, the decision was made to employ xylan xylanase hydrolysis to extract the FOs in BDGs.

### 3.2. Optimization of FO Extraction Process

According to the results of single-factor experiments, the appropriate optimization interval of each single factor was selected (Supplementary Figure S1). The efficiency of xylanase plays a pivotal role in FO extraction, with pH, temperature, time, substrate concentration, and enzyme addition amount selected as critical factors for optimization. A total of 31 experimental sets were conducted in triplicate, with the experimental group order being randomized to minimize design errors. The results are shown in Supplementary Table S3; the absorbance value is the UV absorbance value of each extraction solution and represents the concentration of FOs.

**Figure 2:**
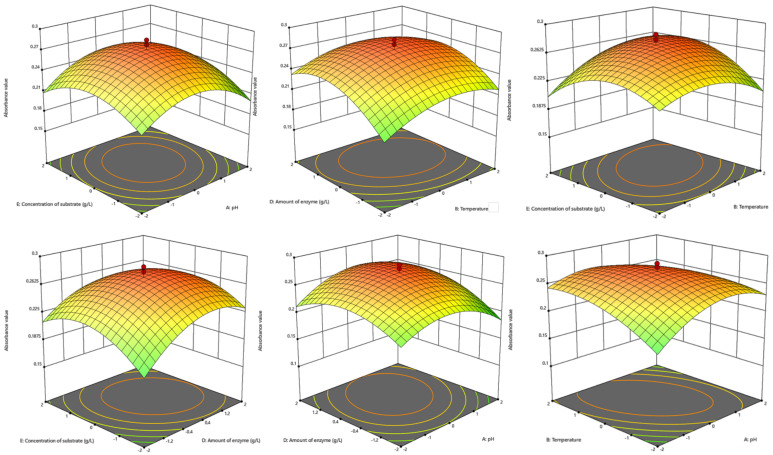
Response surface and contour plot of the FO preparation process optimization.

The regression models underwent comprehensive evaluation through an ANOVA with 95% confidence limits, as depicted in Supplementary Table S4. The regression model exhibited an F value of 42.78, and *p* < 0.0001, signifying its significant impact and its potential for accurate predictions in actual production. The lack of fit term (*p* = 0.2425) and a coefficient of determination R^2^ of 0.9824 indicate that the model is suitable for navigating the design space (Table 1). The Predicted R^2^ of 0.8719 aligns reasonably with the adjusted R^2^ of 0.9595, with the difference being less than 0.2. The Adeq Precision, measuring the signal-to-noise ratio, stands at 21.293, surpassing the desirable ratio of 4, indicating an adequate signal in the regression models (Table 1). Consequently, the fitted model exhibited excellent effectiveness overall and could guide the optimization of the FO preparation process to yield reliable results. The regression coefficients for the primary terms (B, C, D, and E) and secondary terms (AB, AC, AD, BC, BE, CD, and DE) reached significant levels (*p* < 0.05). Evaluating the factors’ influence on absorbance values, the order was found to be C > D > B > E > A based on the F-value. Response surface plots and contour lines revealed that the interaction between pH and temperature had a strong influence, and enzyme amount and substrate concentration significantly impacted FO extraction, as demonstrated in Figure 2.

According to the quadratic regression model derived from response surface analysis, the optimal FO extraction process conditions were predicted to be as follows: pH 4.8, temperature 55.8 °C, time 11.9 h, enzyme addition amount 6.9 g/L, and substrate concentration 121.9 g/L. Considering practical considerations, the final production process conditions were determined to be as follows: pH 5, temperature 55 °C, time 12 h, enzyme addition amount 7 g/L, and substrate concentration 120 g/L.

### 3.3. Characterization of Structure of FOs

**Figure 3:**
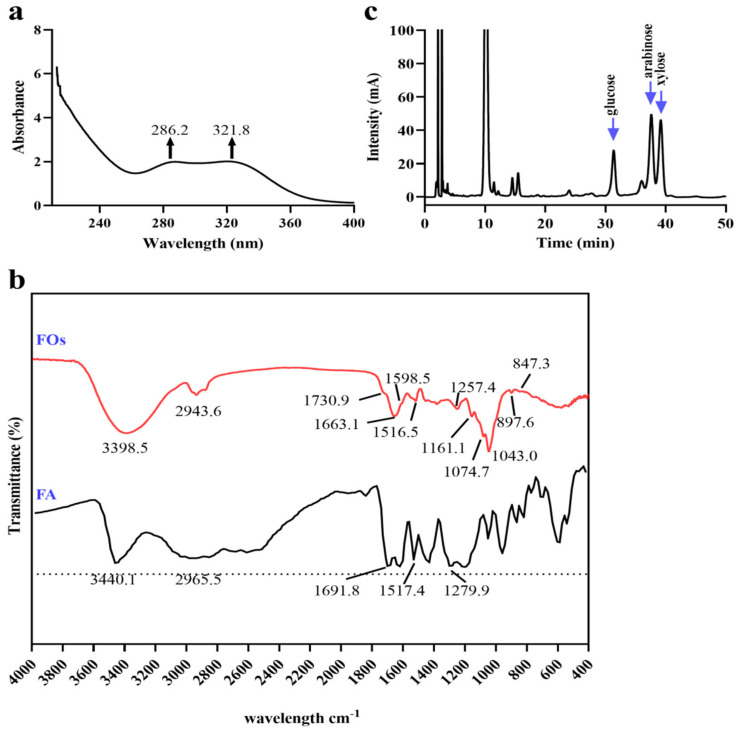
Structural identification of FOs. (**a**) UV absorption chromatogram of FOs; (**b**) FT-IR chromatogram of FOs; (**c**) high-performance liquid chromatography of FO monosaccharide composition.

To achieve a heightened purity of FOs, our post-xylan hydrolysis purification process involved the utilization of XAD-2 macroporous resin. This resin effectively removed polysaccharides while retaining phenolic acids. Subsequent HPLC analysis, as depicted in Figure 1c, confirmed the attained high purity. The determination of the degree of ferulic acid substitution within FOs ensued, with ferulic acid being liberated through an alkali method. According to Table 2, ferulic acid accounted for 5.01% of FOs, which was less than in the FOs extracted from corn bran and more than in the FOs extracted from rice bran [7,26]. The monosaccharide composition of FOs mainly included xylose, arabinose, and glucose (Figure 3c), accounting for 27.22, 24.80, and 15.91%, respectively (Table 2). UV spectral analysis highlighted distinctive characteristics associated with ferulic acid groups. The maximum absorption peak of the ferulic acid standard is at 286 nm, while the maximum absorption peak of FOs falls within the range of 320–325 nm [27]. Figure 3a illustrates the maximum absorption peak of 321.8 nm for FOs, aligning with previous research findings, in lees.

Infrared chromatography, a valuable tool for elucidating structural properties, provided additional insights. Figure 3b displays absorption peaks at 3440.1 cm^−1^, 2925.4 cm^−1^, 1517.4 cm^−1^, and 1279.9 cm^−1^, associated with the -H, -CH, C=O, C=C, and C-O groups on the aromatic ring of ferulic acid [28]. The absorption band at 3398.5 cm^−1^ indicates the stretching of ferulic acid or carbohydrate hydroxyl groups, and the absorption band of 2943.6 cm^−1^ corresponds to the stretching and bending vibrations of C-H [29]. The absorption band of the asymmetrically substituted benzene ring usually occurs around 1600 cm^−1^; hence, 1598.5 cm^−1^ was presumed to be the absorption band of the benzene ring of ferulic acid. The absorption band of 1730.9 cm^−1^ and 1257.4 cm^−1^ are indicative of the ester group between ferulic acid and carbohydrate [30]. The tensile vibration at 1516.5 cm^−1^ may be linked to the degree of substitution of ferulic acid [21,29]. The 1200 cm^−1^ to 900 cm^−1^ region, critical for carbohydrate distinction, exhibits vibrations of C-OH and C-O-C groups [31]. FOs displayed characteristic maximum absorption bands at 1161.1 cm^−1^ and 1043.0 cm^−1^, indicating the presence of xylose oligosaccharides in dianlees. The alterations in the characteristic spectral range of xylose oligosaccharide are associated with the substitution of xylose *O*-3 by arabinose residues, evidenced by absorption bands at 1161.0 cm^−1^ and 1074.7 cm^−1^ [32]. The absorption band at 897.6 cm^−1^ suggests the presence of a β-glycosidic bond due to the ring vibration of C1-H and bending of OH, indicating that xylose residues may be linked by a β-glycosidic bond [33]. The absorption band at 847.3 cm^−1^ indicates the presence of an α-glycosidic bond of the sugar residue [29]. In summary, these findings suggest that the configuration of FOs extracted from BDGs involves a feruloyl arabinosyl group attached to the *O*-3 position of xylose via an α-glycosidic bond, with the xylose group connected by a β-glycosidic bond forming the basic skeleton.

### 3.4. Evaluation of In Vitro Antioxidant Activity of FOs

The in vitro antioxidant assessment serves as a straightforward and highly reproducible method, often employed as an initial screening test for the antioxidant or anti-inflammatory activity of natural extract products. The ABTS and DPPH methods, based on the electron transport mechanism principle, evaluate the antioxidant capacity of compounds by detecting the consumption of free radicals, discerned through the corresponding absorbance changes.

**Figure 4:**
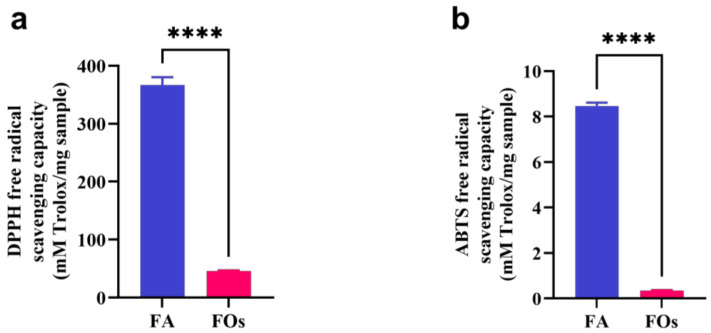
DPPH (**a**) and ABTS (**b**) clear activities of FOs and FA (ferulic acid); the activity was evaluated as the equivalent value of Trolox. The “*” above the columns indicate statistically significant differences, **** means *p* < 0.0001.

In order to better understand the antioxidant ability of FOs primarily based on ferulic acid groups or partly effective oligosaccharides, the experimental concentrations of ferulic acid were designed in proportion to the ferulic acid content in the FOs (FO concentration of one of the 10 points). The results, as illustrated in Figure 4, reveal that the antioxidant capacity of FOs is notably lower than that of ferulic acid. In the DPPH experiment, the equivalent value of ferulic acid is 45.9 ± 0.95 mM Trolox/mg sample, while the equivalent value of FOs is 366.8 ± 10.38 mM Trolox/mg sample (Figure 4a). The substantial difference between ferulic acid and FOs (*p* < 0.0001) indicates that ferulic acid exhibits a significantly higher efficacy in clearing DPPH free radicals than FOs. This disparity may be related to the structure of the DPPH radical and FOs. The DPPH radical has three benzene rings and has a large steric resistance, so a large volume of antioxidants will weaken its reaction with the radical due to steric effect. FOs, comprising arabinose and xylose, in addition to ferulic acid groups, are presumed to have greater steric hindrance compared to ferulic acid. According to the ABTS experiment, the equivalent value of ferulic acid is 8.47 ± 0.11 mM Trolox/mg sample, while the equivalent value of FOs is 0.35 ± 0.01 mM Trolox/mg sample (Figure 4b). The ABTS radical scavenging effect of FOs is significantly lower than that of ferulic acid (*p* < 0.0001), consistent with the results of the DPPH assay.

Despite the experimental concentration of FOs being ten times that of ferulic acid, the antioxidant effect of FOs remains weaker, suggesting a close relationship between the antioxidant ability of FOs and the structure of the contained ferulic acid. Reports of PO-related studies indicate that the antioxidant capacity of synthesized feruloylated oat β-glucan is significantly lower than that of ferulic acid, with increasing feruloyl substitution degrees gradually approaching the antioxidant capacity of ferulic acid [21]. This underscores the pivotal role of ferulic acid in the antioxidant capacity of FOs. Notably, in the DPPH free radical scavenging experiment, the equivalent value of FOs was 366.8 ± 10.38 mM Trolox/mg sample, indicating a stronger antioxidant capacity than Trolox. While FOs may not match the equivalent antioxidant capacity of ferulic acid, they still exhibit robust antioxidant capabilities. However, the lack of biological correlation between the DPPH and ABTS assays emphasizes the need for further studies utilizing biologically relevant models to explore the activity of FOs.

### 3.5. The Alleviating Effect of FOs on LPS-Induced RAW264.7 Cell Inflammation

In order to further explore the biological activity of FOs, a RAW264.7 cell inflammation model was constructed to explore the effect of FOs on the secretion of inflammation-related cytokines. LPS present on Gram-negative bacteria stimulates macrophages to produce nitric oxide (NO) and inflammation-related cytokines.

**Figure 5:**
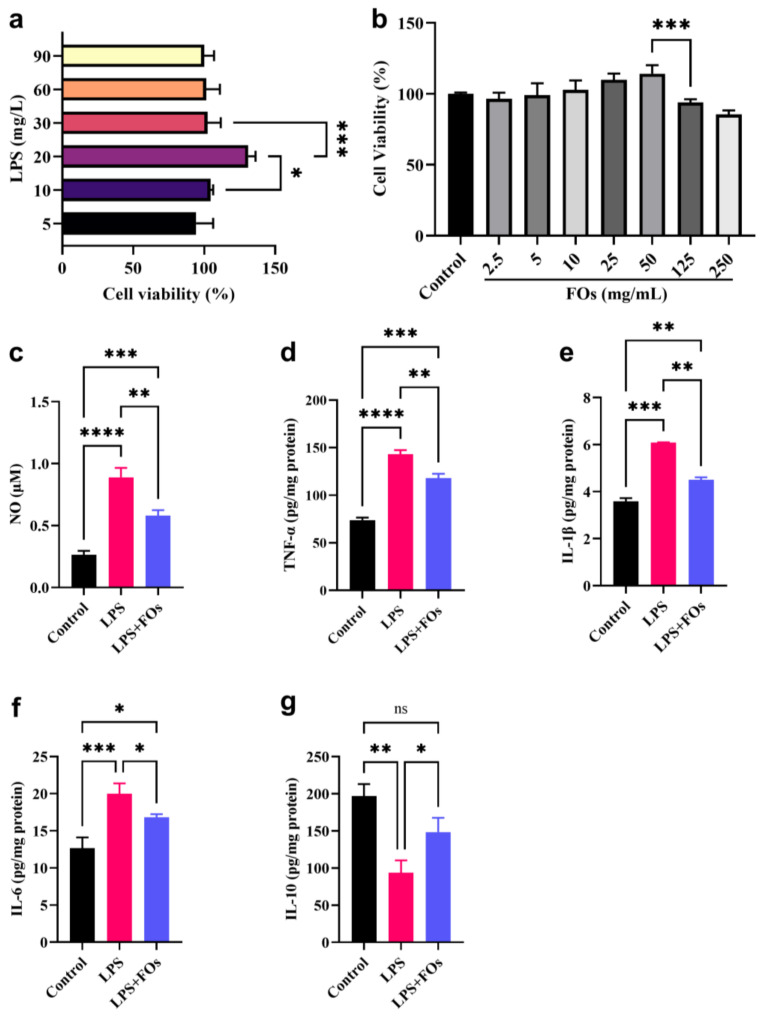
Anti-inflammatory activity of FOs in LPS-induced RAW264.7 inflammation model. (**a**) Optimization of LPS dosage, (**b**) The impact of FOs on the proliferation activity of RAW264.7 cells, (**c**) NO, (**d**) TNF-α, (**e**) IL-1β, (**f**) IL-6, and (**g**) IL-10 in cells. The “*” above the columns indicate statistically significant differences, * means *p* < 0.05; ** means *p* < 0.01; *** means *p* < 0.001; **** means *p* < 0.0001; ns means non-significant differences.

In an effort to optimize LPS concentration for the effective stimulation of macrophages without compromising cell viability, a concentration gradient of LPS was administered. The results, as depicted in Figure 5a, reveal a gradual increase in the activity of RAW264.7 cells with rising LPS concentration, reaching its peak (131%) at 20 mg/L of LPS. Therefore, 20 mg/L of LPS was selected as the optimal concentration for the RAW264.7 cell inflammation model, maximizing macrophage activity and immune function. Furthermore, in the optimization experiment for FO dosing, as shown in Figure 5b, RAW264.7 cell viability reached its peak when the concentration of FOs reached 50 mg/mL, while at 125 mg/mL, cell viability sharply declined. Therefore, a concentration of 50 mg/mL was chosen as the dosing concentration for FOs in subsequent experiments.

NO secretion serves as an indicator of overall macrophage inflammation severity. Figure 5c illustrates the results of NO determination, demonstrating that LPS administration significantly enhanced NO secretion in RAW264.7 cells compared to the control group (*p* < 0.0001). However, in the presence of FOs, the secretion of NO was significantly reduced (*p* < 0.01), indicating the effective inhibitory action of FOs on LPS-stimulated NO secretion in RAW264.7 cells.

Macrophages play a key role in the normal inflammatory response or immune response by secreting cytokines such as TNF-α, IL-6, IL-1β, and IL-10. TNF-α induces the further secretion of IL-1β and IL-6, exacerbating inflammation and leading to cell apoptosis. IL-10 is an important anti-inflammatory cytokine and can inhibit the expression of TNF-α and IL-1β. The results of our determination of inflammatory factors in RAW264.7 cells, presented in Figure 5d–g, demonstrate that LPS significantly promotes the secretion of TNF-α, IL-6, and IL-1β relative to the control group (*p* < 0.001). FO administration significantly inhibits these pro-inflammatory factors (*p* < 0.05), although the levels of inflammatory factors in the FO group do not fully revert to the control levels. Regarding IL-10, LPS notably suppresses its secretion compared to the control group (*p* < 0.05), while FO administration significantly enhances IL-10 secretion (*p* < 0.05), even restoring it to the control group level (*p* > 0.05). This suggests that FOs not only inhibit the secretion of pro-inflammatory factors but also promote the secretion of anti-inflammatory factors. In conclusion, FOs significantly suppress the LPS-induced inflammatory response in RAW264.7 cells.

## 4. Discussion

The primary component of the BDGs utilized in this study was sorghum husk, and the preliminary determination of the FO structure suggested similarity to that of corn bran and wheat bran. While the activities or physical properties of FOs may be comparable due to this structural resemblance, there is a paucity of research on FOs in distillers’ grains. After the microbial fermentation of BDGs, in addition to the complete utilization of starch, microorganisms also secrete various enzymes such as cellulase and protease [34,35,36]. This microbial activity contributes to the decomposition of sorghum shell and other raw materials in distillers’ grains, potentially enhancing the extraction of FOs. Additionally, the initial low pH of distillers’ grains (approximately 4) is conducive to enzymes that thrive in acidic environments, such as xylanase. The direct extraction of FOs using enzymes can save energy and eliminate the need for adding acidic chemicals to adjust the pH, reducing the environmental burden. Consequently, FO extraction was selected directly in the optimization process of the response surface in this study.

While in vitro antioxidant activity experiments provide insights into the direct antioxidant capacity of FOs against free radicals, it is essential to consider additional factors in practical scenarios. Unlike ferulic acid, which can be directly absorbed by the blood, FOs require microbial fermentation in the large intestine to release ferulic acid and oligosaccharides [37]. As a result, FOs exhibit a longer half-life, and the further fermentation of oligosaccharides can regulate intestinal microorganisms, contributing to a more sustained and active role. Therefore, FOs may offer enhanced antioxidant activity in practical applications. Furthermore, phenolic acid–polysaccharide conjugates have the potential to address the limitations associated with the water solubility, low bioavailability, and rapid catabolism of phenolic acids [1]. They can also enhance the antioxidant activity of polysaccharides, thereby offering promising avenues for the application of FOs.

Inflammation serves as a protective mechanism in the human body against external stimuli. However, the excessive secretion of inflammatory factors can lead to immune imbalance, posing risks to human health. TNF-α, IL-6, and IL-1β are pivotal inflammatory factors implicated in the inflammatory process capable of initiating cascading reactions within the immune system and influencing multiple inflammatory pathways [38]. Hence, we chose to investigate the impact of FOs on the secretion of these three inflammatory factors in RAW264.7 cells. The study results demonstrate that FOs derived from BDGs effectively inhibit the secretion of these pro-inflammatory factors in macrophages. Concurrently, the promotion of IL-10 secretion by FOs can negatively regulate the secretion of pro-inflammatory factors, indicating a potential anti-inflammatory effect. Considering the metabolic properties of FOs, which are metabolized in the human large intestine, FOs from distillers’ grains may have a mitigating effect on inflammatory bowel disease. Furthermore, the administration of 100 μg/mL of FOs derived from rice bran demonstrated a reduction in the expression levels of TNF-α and NO in RAW264.7 cells [39], consistent with the findings of this study. Additionally, upon entering the intestine, FOs have the potential to undergo metabolism by ferulic acid esterase secreted by gut microorganisms, leading to the liberation of free ferulic acid [5]. Consequently, both ferulic acid residues and polysaccharides may exert immune-regulatory effects. However, a comprehensive understanding of the combined action of these components necessitates further investigation through animal experiments and metabolic studies.

## 5. Conclusions

In summary, the POs found in BDG are FOs, with their primary structure being ferulic acid esterified onto arabinoxylan oligosaccharides, exhibiting a ferulic acid substitution degree of 5.01, and the FO extraction process was optimized using a central composite design. FOs in BDGs demonstrate a significant in vitro free radical scavenging capacity. However, even at one-tenth of the dosage of FOs, ferulic acid exhibits greater in vitro antioxidant activity than FOs, indicating a close correlation between the antioxidant activity of FOs and the presence of ferulic acid residues. Furthermore, FOs can significantly inhibit the secretion of pro-inflammatory cytokines (IL-6, IL-1β, TNF-α) and promote the secretion of the anti-inflammatory cytokine (IL-10) induced by LPS in RAW264.7 cells. Lastly, this study provides new methods for the high-value utilization and processing of distillers’ grains in the baijiu industry and offers new references for the development of health products related to FOs.

## Figures and Tables

**Table 1:** Fit statistics of the model.

Categories of Statistics	Numerical Value
R^2^	0.9824
Adjusted R^2^	0.9595
Predicted R^2^	0.8719
Adeq Precision	21.2933

**Table 2:** The monosaccharide composition of FOs and the degree of substitution of ferulic acid.

**Monosaccharide Residue**	**Mass (µg)**	**Mol %**
Mannose (Man)	27.63	2.76
Rhamnose (Rha)	3.15	0.32
Glucuronic acid (GlcA)	22.14	2.21
Galacturonic acid (GalA)	12.33	1.23
Glucose (Glc)	159.12	15.91
Galactose (Gal)	41.67	4.17
Xylose (Xyl)	272.16	27.22
Arabinose (Ara)	248.04	24.80
Fucose (Fuc)	5.4	0.54
Total	791.73	79.17
**Phenolic Acid Species**	**Mass (µg)**	**Degree of Substitution**
Ferulic acid	39.69	5.01

## Data Availability

The original contributions presented in the study are included in the article/Supplementary Materials, further inquiries can be directed to the corresponding author.

## Supplementary Materials

The following supporting information can be downloaded at: https://www.mdpi.com/2304-8158/13/8/1283/s1. Figure S1: Results of five single-factor experiments. (a) pH; (b) time; (c), substrate concentration; (d) enzyme addition amount; (e) temperature. Table S1: Standard curve of mixed monosaccharide standards. Table S2: Variables and their levels employed in a central composite rotatable design. Table S3: Experimental design matrix and results. Table S4: ANOVA for reduced quadratic model.
