# Supplementary material for: Extraction, Identification, and In Vitro Anti-Inflammatory Activity of Feruloylated Oligosaccharides from Baijiu Distillers’ Grains
Source: Foods. 2024 Apr 22;13(8):1283. doi: 10.3390/foods13081283 (PMC11049520; doi:10.3390/foods13081283)
Supplement: Supplementary file 1 [file foods-13-01283-s001.zip › Supplementary Material.pdf]

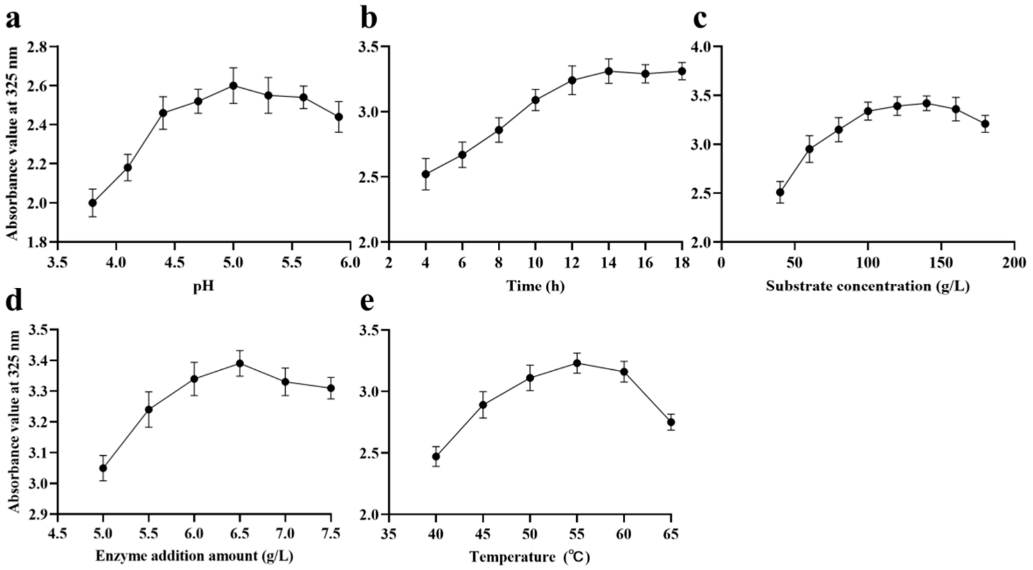

Supplementary Figure S1. Results of five single factor experiments. (a) pH; (b) time; (c), substrate concentration; (d) enzyme addition amount; (e) temperature.

Supplementary Table S1. Standard curve of mixed monosaccharide standards.

Monosaccharides	Regression equation	Linear range (ug/mL)	R^2	LOD (ug/mL)	LOQ (ug/mL)
Mannose	y=30.137x+4932	0.2 - 500.0	0.9924	0.04	0.15
Ribose	y=31.156x+2605	0.2 - 500.0	0.9949	0.16	0.44
Rhamnose	y=18.221x+1836	0.2 - 500.0	0.9915	0.09	0.36
Glucuronic acid	y=23.062x+2958	0.2 - 500.0	0.9992	0.05	0.21
Galacturonic acid	y=24.271x+13923	0.2 - 500.0	0.9963	0.14	0.41
N-acetylglucosamine	y=19.441x+7826	0.2 - 500.0	0.9958	0.06	0.18
Glucose	y=28.963x+24264	0.2 - 500.0	0.9991	0.02	0.11
N-acetylgalactosamine	y=23.713x+15304	0.2 - 500.0	0.9935	0.11	0.39
Galactose	y=35.191x+57034	0.2 - 500.0	0.9946	0.05	0.17
Xylose	y=35.673x+32958	0.2 - 500.0	0.9961	0.08	0.28
Arabinose	y=38.091x+3493	0.2 - 500.0	0.9982	0.13	0.42
Fucose	y=28.085x+24859	0.2 - 500.0	0.9978	0.17	0.55

Supplementary Table S2. Variables and their levels employed in a central composite rotatable design.

Variable	Coded levels				
	-2	-1	0	1	2
pH	4	4.5	5	5.5	6
Temperature (°C)	45	50	55	60	65
Time (h)	10	12	14	16	18
Substrate concentration (g/L)	80	100	120	140	160
Enzyme addition amount (g/L)	5.5	6	6.5	7	7.5

Supplementary Table S3. Experimental design matrix and results.

Run	A:pH	B:Temperature (°C)	C:Time (h)	D:Amount of enzyme (g/L)	E:Concentration of substrate (g/L)	Response: Absorbance value
1	2	2	2	2	2	0.2528
2	-2	2	2	2	-2	0.2162
3	-2	-2	-2	-2	2	0.1216
4	2	2	-2	-2	2	0.1203
5	0	0	-4	0	0	0.1851
6	2	-2	2	2	-2	0.2440
7	2	-2	2	-2	2	0.1502
8	0	0	0	0	0	0.2702
9	0	4	0	0	0	0.2015
10	2	2	-2	2	-2	0.0861
11	0	0	0	-4	0	0.1189
12	0	0	0	0	0	0.2794
13	-4	0	0	0	0	0.1259
14	-2	2	-2	2	2	0.1474
15	0	0	0	0	0	0.2794
16	0	0	0	0	0	0.2622
17	-2	-2	2	-2	-2	0.1138
18	-2	2	2	-2	2	0.2188
19	-2	-2	2	2	2	0.1254
20	2	2	2	-2	-2	0.1283
21	0	0	4	0	0	0.2844
22	0	-4	0	0	0	0.1611
23	0	0	0	0	0	0.2861
24	4	0	0	0	0	0.1269
25	-2	-2	-2	2	-2	0.1442
26	0	0	0	0	-4	0.1386
27	0	0	0	4	0	0.1776
28	0	0	0	0	4	0.1630
29	2	-2	-2	2	2	0.1566
30	-2	2	-2	-2	-2	0.1340
31	2	-2	-2	-2	-2	0.1337

Supplementary Table S4. ANOVA for reduced quadratic model.

Source	Sum of Squares	df	Mean Square	F-value	p-value	
Model	0.1128	17	0.0066	42.78	< 0.0001	significant
A-pH	0.0001	1	0.0001	0.6105	0.4486	
B-Temperature	0.0017	1	0.0017	10.76	0.0060	
C-Time	0.0150	1	0.0150	96.43	< 0.0001	
D-Amount of enzyme	0.0055	1	0.0055	35.70	< 0.0001	
E-Concentration of substrate	0.0008	1	0.0008	5.01	0.0434	
AB	0.0061	1	0.0061	39.59	< 0.0001	
AC	0.0015	1	0.0015	9.89	0.0077	
AD	0.0017	1	0.0017	11.22	0.0052	
BC	0.0038	1	0.0038	24.36	0.0003	
BE	0.0040	1	0.0040	25.55	0.0002	
CD	0.0027	1	0.0027	17.38	0.0011	
DE	0.0007	1	0.0007	4.40	0.0560	
A^2	0.0336	1	0.0336	216.34	< 0.0001	
B^2	0.0122	1	0.0122	78.47	< 0.0001	
C^2	0.0015	1	0.0015	9.98	0.0075	
D^2	0.0238	1	0.0238	153.30	< 0.0001	
E^2	0.0228	1	0.0228	146.73	< 0.0001	
Residual	0.0020	13	0.0002			
Lack of Fit	0.0017	9	0.0002	2.13	0.2425	not significant
Pure Error	0.0003	4	0.0001			
Cor Total	0.1148	30				
